# Improving the Methanol Tolerance of an *Escherichia coli* Methylotroph via Adaptive Laboratory Evolution Enhances Synthetic Methanol Utilization

**DOI:** 10.3389/fmicb.2021.638426

**Published:** 2021-02-11

**Authors:** R. Kyle Bennett, Gwendolyn J. Gregory, Jacqueline E. Gonzalez, Jie Ren Gerald Har, Maciek R. Antoniewicz, Eleftherios T. Papoutsakis

**Affiliations:** ^1^Department of Chemical and Biomolecular Engineering, University of Delaware, Newark, DE, United States; ^2^Molecular Biotechnology Laboratory, The Delaware Biotechnology Institute, University of Delaware, Newark, DE, United States

**Keywords:** synthetic methylotrophy, methanol tolerance, *E. coli*, methanol, methanol toxicity

## Abstract

There is great interest in developing synthetic methylotrophs that harbor methane and methanol utilization pathways in heterologous hosts such as *Escherichia coli* for industrial bioconversion of one-carbon compounds. While there are recent reports that describe the successful engineering of synthetic methylotrophs, additional efforts are required to achieve the robust methylotrophic phenotypes required for industrial realization. Here, we address an important issue of synthetic methylotrophy in *E. coli*: methanol toxicity. Both methanol, and its oxidation product, formaldehyde, are cytotoxic to cells. Methanol alters the fluidity and biological properties of cellular membranes while formaldehyde reacts readily with proteins and nucleic acids. Thus, efforts to enhance the methanol tolerance of synthetic methylotrophs are important. Here, adaptive laboratory evolution was performed to improve the methanol tolerance of several *E. coli* strains, both methylotrophic and non-methylotrophic. Serial batch passaging in rich medium containing toxic methanol concentrations yielded clones exhibiting improved methanol tolerance. In several cases, these evolved clones exhibited a > 50% improvement in growth rate and biomass yield in the presence of high methanol concentrations compared to the respective parental strains. Importantly, one evolved clone exhibited a two to threefold improvement in the methanol utilization phenotype, as determined via ^13^C-labeling, at non-toxic, industrially relevant methanol concentrations compared to the respective parental strain. Whole genome sequencing was performed to identify causative mutations contributing to methanol tolerance. Common mutations were identified in 30S ribosomal subunit proteins, which increased translational accuracy and provided insight into a novel methanol tolerance mechanism. This study addresses an important issue of synthetic methylotrophy in *E. coli* and provides insight as to how methanol toxicity can be alleviated via enhancing methanol tolerance. Coupled improvement of methanol tolerance and synthetic methanol utilization is an important advancement for the field of synthetic methylotrophy.

## Introduction

There is great interest in utilizing methane and methanol from natural gas reserves as industrial feedstocks (Haynes and Gonzalez, 2014). Product yields from methane and methanol can be increased due to the fact that they more reduced than carbohydrates (Whitaker et al., 2015). Furthermore, biological conversion of methane and methanol is preferable to chemical catalysis, as it does not require extreme conditions and has higher specificity. Native methylotrophs are not as genetically tractable and have much slower growth kinetics than established platform hosts such as *E. coli* (Bennett et al., 2018b). Therefore, emphasis has been placed on the development and utilization of synthetic methylotrophic organisms that are engineered to utilize methane and methanol (Whitaker et al., 2015; Bennett et al., 2018b).

**FIGURE 1:**
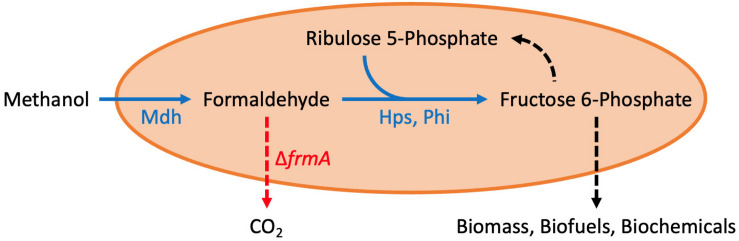
Synthetic methanol metabolism in *E. coli*. Methanol dehydrogenase (mdh), hexulose phosphate synthase (hps), phosphohexulose isomerase (phi), and formaldehyde dissimilation pathway (*frmRAB*). See text for more details. This figure was adapted from Bennett et al. (2020a).

In the last several years, there have been multiple approaches to engineer synthetic methylotrophs, with most work done in *E. coli* (Price et al., 2016; Bennett et al., 2018a, 2020c; Chen et al., 2018; Gonzalez et al., 2018; Meyer et al., 2018; Woolston et al., 2018a; Zhang et al., 2018; Rohlhill et al., 2020), *Corynebacterium glutamicum* (Lessmeier et al., 2015; Witthoff et al., 2015; Tuyishime et al., 2018), and *Saccharomyces cerevisiae* (Dai et al., 2017). As illustrated in Figure 1, the ribulose monophosphate (RuMP) pathway is the most often employed pathway for synthetic methylotrophy. This pathway is made up of two enzymes, Hps (hexulose phosphate synthase) and Phi (phosphohexulose isomerase). Together with Mdh (methanol dehydrogenase), this pathway oxidizes methanol to formaldehyde (via Mdh), which is then fixed with ribulose 5-phosphate to produce hexulose 6-phosphate (via Hps), which is finally converted to fructose 6-phosphate (via Phi).

Autonomous synthetic methylotrophy, wherein growth on methane or methanol does not require additional carbon sources, has been difficult to realize. Only one study to date has reported the successful construction of a true *E. coli* methylotroph (Chen et al., 2020). In order to achieve this feat, the authors relied on a combination of rational engineering and adaptive laboratory evolution (ALE). The resulting evolved strain exhibited a doubling time of 8.5 h and was able to grow to an optical density (OD) of 2 in methanol minimal medium. Optimal growth was observed at methanol concentrations between 400 and 600 mM. Growth defects became obvious at 1 M methanol, and growth was completely abolished at 1.5 M methanol, highlighting the negative consequences of methanol toxicity and need for improved methanol tolerance in order to achieve a more robust phenotype.

The difficulty in construction of true synthetic methylotrophs has been attributed to multiple causes, including poor enzyme kinetics (Wu et al., 2016; Roth et al., 2019), the inability to remove a native co-substrate due to growth dependence (Chen et al., 2018; Meyer et al., 2018; Antoniewicz, 2019), a lack of proper gene regulation in synthetic hosts (Rohlhill et al., 2017, 2020; Woolston et al., 2018b), poor synthesis of proteinogenic amino acids from methanol carbon (Gonzalez et al., 2018), and the cytotoxicity of methanol (Lessmeier and Wendisch, 2015; Wang et al., 2020). The cytotoxicity of methanol is twofold: methanol alters the fluidity and biological properties of cellular membranes (Gustafson and Tagesson, 1985; Sonmez et al., 2013) while formaldehyde, the oxidation product of methanol, reacts readily with proteins and DNA (Chang and Gershwin, 1992; Teng et al., 2001). Due to these cytotoxic effects, there is interest to improve the methanol and/or formaldehyde tolerance of native and synthetic methylotrophs. It has been demonstrated that *E. coli* tolerates methanol relatively well, growing in the presence of 4% (v/v), or ca. 1 M, methanol in Luria-Bertani (LB) media (Ganske and Bornscheuer, 2006). However, the same study reported complete growth inhibition at a methanol concentration of 10% (v/v), or ca. 2.5 M. Improved methanol tolerance is not only beneficial for methylotrophic *E. coli*, but also lends itself to fermentations where the substrates, media components or fermentation conditions contain methanol as an impurity. For example, crude glycerol may contain methanol as an impurity depending upon how it is processed (Yang et al., 2012).

Formaldehyde is a more potent cytotoxin to cells than methanol due to its high reactivity with nucleic acids and proteins, which results in cross-linking (Chang and Gershwin, 1992; Teng et al., 2001). Therefore, in addition to methanol’s direct effect on cells, it also indirectly impacts cells via its oxidation product. Formaldehyde must therefore be assimilated or dissimilated readily. In *E. coli*, a linear dissimilation pathway, encoded by the *frmRAB* operon, exists to combat endogenous formaldehyde resulting from select metabolic pathways or oxidative demethylation of nucleic acids (Gonzalez et al., 2006). Specifically, formaldehyde induces expression of this operon via a formaldehyde-responsive promoter, which is transcriptionally regulated by the repressor *frmR* (Gonzalez et al., 2006; Rohlhill et al., 2017). In a two-step process, formaldehyde is readily oxidized to formate by *S*-hydroxymethylglutathione dehydrogenase (*frmA*) and *S*-formylglutathione hydrolase (*frmB*). Formate can then be further oxidized to CO_2_. However, many studies geared toward engineering synthetic methylotrophs have relied on a Δ*frmA* genetic background in order to conserve formaldehyde carbon for assimilation to support methylotrophic growth (Muller et al., 2015; Whitaker et al., 2015). Thus, the need for improved methanol/formaldehyde tolerance becomes more apparent when this “safety valve” is removed from the cell. In order to utilize formaldehyde for cell growth, native and synthetic methylotrophs must contain a formaldehyde assimilation pathway to capture carbon and energy from formaldehyde while alleviating toxicity. As described above, the RuMP pathway is of considerable interest for engineering synthetic methylotrophs since it is the most energy efficient pathway among the three candidates and requires only two core enzymes (Whitaker et al., 2015).

Efforts to improve the methanol tolerance of native and synthetic methylotrophs have been reported for *B. methanolicus* and *C. glutamicum*. For the *B. methanolicus* study, it was reported that upregulation of genes involved in methanol oxidation and the RuMP pathway contributed to improved methanol and formaldehyde tolerance (Jakobsen et al., 2006). For one *C. glutamicum* study, ALE was performed on a non-methylotrophic *C. glutamicum* wild-type strain, which resulted in an evolved clone that was more tolerant to all methanol concentrations up to 3 M (Lessmeier and Wendisch, 2015). A second *C. glutamicum* study improved the methanol tolerance of a methylotrophic *C. glutamicum* methanol auxotroph, which resulted in an evolved strain that could tolerate up to 20 g/L (ca. 600 mM) methanol without any growth defects (Tuyishime et al., 2018; Wang et al., 2020).

Collectively, these previous studies highlight the many direct and indirect effects of methanol toxicity and various ways that methanol tolerance can be achieved. In this study, we performed ALE to improve the methanol tolerance of several *E. coli* strains, both methylotrophic and non-methylotrophic, and used WGS analysis to identify the common mutations responsible for methanol tolerance. Our results provide insight into a novel mechanism for methanol tolerance, which occurs from mutations in 30S ribosomal subunit proteins, and emphasize the ability to couple improved methanol tolerance with enhanced synthetic methanol utilization.

## Materials and Methods

### Chemicals

All chemicals were purchased from Sigma-Aldrich (St. Louis, MO) unless noted otherwise. ^13^C-methanol (99% ^13^C) was purchased from Cambridge Isotope Laboratories (Tewksbury, MA). *E. coli* NEB5α, Q5 DNA polymerase and NEBuilder HiFi DNA assembly master mix were purchased from NEB (Ipswich, MA). Restriction endonucleases were purchased from Thermo Fisher Scientific (Waltham, MA).

### Strains and Plasmids

All strains, plasmids, and primers used in this study are listed in Supplementary Tables S1–S3, respectively. *E. coli* NEB5a was used for plasmid construction and propagation. *E. coli* BW25113 D*frmA* was obtained from the Keio collection and used for growth characterization (Baba et al., 2006). Deletion of *ihfA* was performed as previously described via the lambda red recombineering system (Datsenko and Wanner, 2000; Bennett et al., 2020a). Methanol assimilation genes were cloned into pETM6 (Xu et al., 2012) for episomal expression as previously described to produce the pUD9 plasmid (Bennett et al., 2018a). Overexpression of *crp* was achieved as previously described (Bennett et al., 2020a).

### Media and Growth Conditions

*E. coli* strains were routinely cultured in LB medium supplemented with the appropriate antibiotics (100 μg/mL ampicillin, 25 μg/mL kanamycin) unless otherwise noted. Growth characterization of *E. coli* strains for methanol or formaldehyde tolerance was performed in 250 mL baffled flasks containing 30 mL LB medium supplemented with methanol or formaldehyde at the specified concentrations at 37°C and 250 RPM. Cell growth rate was determined every hour as follows: ln(C/C_0_)/(t − t_0_), where C and C_0_ represent the biomass concentration at the current (t) and prior (t_0_) times (e.g., 3 and 2 h). The highest value was selected as the maximum growth rate. For methylotrophic growth and ^13^C-labeling assays, an overnight culture of the respective *E. coli* strain in LB medium was used to inoculate fresh M9 minimal medium containing 1 g/L yeast extract with or without 60 mM ^13^C-methanol to an OD_600_ of approximately 0.05. Samples were collected at 48h for labeling analysis.

### Chemical Mutagenesis and Adaptive Laboratory Evolution (ALE)

Chemical mutagenesis was performed as described previously (Sandoval et al., 2015). Briefly, 70 μL of an overnight culture of *E. coli* Δ*frmA* pUD9 was used to inoculate 7 mL of fresh LB medium. Cells were grown aerobically at 37°C until an OD_600_ of 1. Cells were then harvested via centrifugation (4,000 g, 10 min), washed once with 10 mL of PT buffer (0.1 g/L peptone, 8.5 g/L sodium chloride, 1 g/L sodium thioglycolate), harvested again via centrifugation (4,000 g, 10 min) and finally resuspended in 7 mL of fresh LB medium. 200 μL of 2.5 g/L *N*-methyl-*N’*-nitro-*N*-nitrosoguanidine (NTG) was then added, followed by a 20 min incubation at 37°C. Mutagenized cells were then harvested via centrifugation (4,000 g, 10 min) and washed thrice with 10 mL of PT buffer, followed by resuspension in 10 mL of fresh LB medium and outgrowth overnight at 37°C. The lethality of this chemical mutagenesis was determined to be ca. 99% as CFUs/mL directly prior to NTG treatment were ca. 7.2 × 10^8^ and ca. 6.4 × 10^4^ directly following NTG treatment.

After overnight recovery, mutagenized cells were subjected to directed evolution via passaging in fresh LB medium supplemented with methanol. After the initial recovery, cells were used to inoculate fresh LB medium supplemented with 1 M methanol. Upon growth of this culture, cells were used to inoculate fresh LB medium supplemented with 1.25 M methanol. This procedure was continued for 1.5, 1.75, and 2 M methanol. Upon growth in fresh LB medium supplemented 2 M methanol, a frozen stock in 20% glycerol was made. This frozen stock was streaked on a fresh LB agar plate to isolate individual clones. Six of these clones were analyzed for improved methanol tolerance over the non-evolved parent strain in fresh LB medium supplemented with 2 M methanol. The clone exhibiting the most improved methanol tolerance was used for further analysis.

Three other *E. coli* strains (Δ*frmA*, Δ*frmA*Δ*ihfA* + pUD9 and Δ*frmA* + pUD9 + pCrp) were also subjected to ALE without NTG mutagenesis. Serial batch passaging in LB medium supplemented with increasing methanol concentrations was performed in a similar manner until methanol-tolerant clones could be isolated. Approximately 10 passages were required to achieve improved methanol tolerance when chemical mutagenesis was not used.

### Resting Cell Assays

Mdh, Hps and Phi *in vivo* assays were performed as described (Whitaker et al., 2017). Briefly, *E. coli* Δ*frmA* strains expressing *B. stearothermophilus* Mdh and *B. methanolicus* Hps and Phi were grown from a colony in LB for 6 h at 37°C with shaking (225 rpm). Cells were then washed twice in M9 minimal medium and adjusted to an OD_600_ of 1.0 in M9 minimal medium. Methanol was added to a final concentration of 1 M while formaldehyde was added to a final concentration of 1 mM. At the indicated time points, samples were collected and 400 μL of culture supernatant was mixed with 800 μL of Nash reagent to assay for formaldehyde concentration (Nash, 1953).

### Analytical Methods

Biomass concentration was determined as previously described (Whitaker et al., 2017; Bennett et al., 2018a). Briefly, OD_600_ was measured on a Beckman-Coulter DU730 spectrophotometer. Methanol boost was calculated as the percentage improvement of biomass yield of a culture in the presence of methanol as compared to the control without methanol (Whitaker et al., 2017; Bennett et al.,2020a,b). Methanol was measured via high performance liquid chromatography (HPLC) (Whitaker et al., 2017). Extraction of metabolites and proteinogenic amino acids was performed as previously described and analyzed for ^13^C-labeling using gas chromatography-mass spectrometry (Whitaker et al., 2017; Bennett et al., 2018a, 2020a, 2020b,c; Long and Antoniewicz, 2019). ^13^C-labeling was determined from the measured mass isotopomer data (Whitaker et al., 2017; Long and Antoniewicz, 2019). Statistics were calculated using a two-tailed unpaired *t*-test with a 95% confidence interval.

### Whole Genome Sequencing

Whole genome sequencing of Δ*frmA* parental and evolved strains, Δ*ihfA* parental and evolved strains, and KB201, a Δ*frmA* strain that was subjected to directed evolution without the pUD9 plasmid, was performed as previously described (Bennett et al., 2020c). Briefly, genomic DNA was extracted using a Qiagen DNeasy Blood and tissue kit per manufacturer’s protocol (Germantown, MD). Genomic DNA was then sequenced on an RSII sequencer system (Pacific Biosciences, Menlo Park, CA) using single molecule, real time (SMRT) sequencing (University of Delaware DNA Sequencing and Genotyping Center), with average read length of 10 kb generated. Sequencing analysis was performed with the SMRT Link software via the resequencing application (Pacific Biosciences). *E. coli* BW25113 (GenBank CP009273.1) was used as the reference genome. Mutations unique to each sequenced strain in comparison to the respective parental strain were chosen.

## Results

### Adaptive Laboratory Evolution, Combined With Chemical Mutagenesis, Enhances the Methanol Tolerance of a Synthetic *E. coli* Methylotroph

**FIGURE 2:**
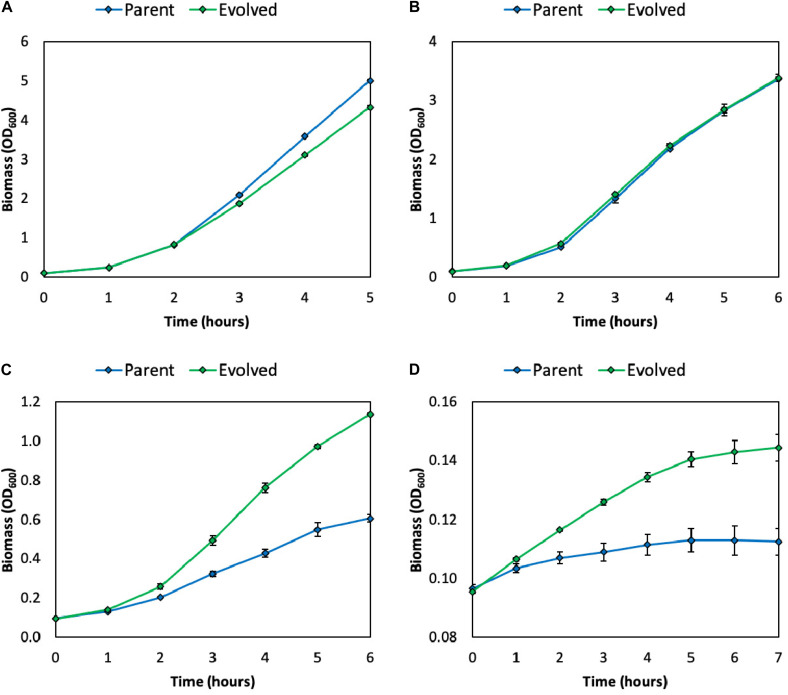
Growth of parental and evolved methylotrophic *E. coli* strains in LB medium supplemented with 0 **(A)**, 1 **(B)**, 2 **(C)**, or 3 **(D)** M methanol. Error bars indicate standard error (*n* = 2).

As discussed, methanol and formaldehyde are cytotoxic to cells. In LB medium, the growth rate of a synthetic *E. coli* methylotroph (*E. coli* BW25113 Δ*frmA* + pUD9) is severely inhibited above methanol concentrations of 1 M (Figure 2 and Supplementary Figure S1A). Specifically, the growth rates in the presence of 0, 1, 2, and 3 M methanol are 1.2 ± 0.02, 1.0 ± 0.01, 0.47 ± 0.02, and 0.07 ± 0.00 h^–1^, respectively. Since several studies have reported using high methanol concentrations, specifically ≥ 1 M, for natural and synthetic methylotrophs, specifically *B. methanolicus* (Bozdag et al., 2015) and *E. coli* (Muller et al., 2015), improved methanol tolerance at these high concentrations would be beneficial.

To improve the methanol tolerance of this synthetic *E. coli* methylotroph (*E. coli* BW25113 Δ*frmA* + pUD9), chemical mutagenesis and ALE were performed. Briefly, cells were first mutagenized with *N*-methyl-*N’*-nitro-*N*-nitrosoguanidine (NTG), which mutates DNA by alkylating guanine and thymine, resulting in transition mutations between GC and AT, recovered overnight in LB medium and then subjected to several rounds of passaging in LB medium supplemented with increasing methanol concentrations (Supplementary Figure S1B). After outgrowth in the presence of 2 M methanol, a frozen stock was prepared and subsequently streaked onto an LB agar plate to isolate individual clones. Six clones were examined for improved growth in LB medium supplemented with 2 M methanol (Supplementary Figure S2). One of these clones, “Evolved 3,” simply referred to as “evolved,” exhibited the largest improvement in growth and was selected for further analysis. Indeed, this evolved clone exhibited improved methanol tolerance at high methanol concentrations, i.e., 2–3 M (Figures 2C,D and Supplementary Figure S1A). Specifically, the growth rates in the presence of 0, 1, 2, and 3 M methanol were 1.2 ± 0.02, 1.1 ± 0.00, 0.65 ± 0.01, and 0.11 ± 0.01 h^–1^, respectively (Supplementary Table S4). Thus, the evolved clone exhibited growth rate improvements of 10, 38, and 57% over the parental strain in 1, 2, and 3 M methanol, respectively. Additionally, the evolved clone achieved higher final biomass titers over the parent strain in LB medium supplemented with 2 and 3 M methanol (Figures 2C,D). Taken together, these results demonstrate the usefulness of chemical mutagenesis and ALE for improving tolerance to toxic substrates.

### Methanol Tolerance of the Evolved Clone Is Specific to Methanol, Not Formaldehyde

To investigate whether the improved methanol tolerance of the evolved clone resulted from improved methanol and/or formaldehyde tolerance, the parental strain and evolved clone were cured of the pUD9 plasmid via serial passaging in the absence of the appropriate antibiotic so that formaldehyde tolerance of both strains could be determined. Plasmid curing was essential for this since both the Mdh and RuMP pathway enzymes readily consume formaldehyde, either via reducing it back to methanol or assimilating it into central carbon metabolism, respectively. Both pathways result in inaccurate growth rate measurements since the formaldehyde concentration is continually decreasing to non-toxic levels over time, thus yielding a dynamic growth rate (Supplementary Figure S3). Furthermore, the evolved, plasmid-containing clone was not observed to overcome formaldehyde toxicity more quickly than the plasmid-containing parental strain (Supplementary Figure S3), suggesting that the observed methanol tolerance of the evolved clone results from improved tolerance to methanol and not formaldehyde.

The resulting growth rates of both plasmid-cured strains in LB medium supplemented with varying levels of formaldehyde were similar (Supplementary Figures S4, S5), again suggesting that the observed methanol tolerance of the evolved clone results from improved tolerance to methanol and not formaldehyde, at least at the concentrations tested in this study. Furthermore, as discussed, formaldehyde exerts greater cytotoxicity on cells than does methanol, as indicated by the severe reduction in growth rate at low (i.e., mM) formaldehyde concentrations compared to high (i.e., M) methanol concentrations. Specifically, the growth rates of the plasmid-cured parental strain in the presence of 0, 0.25, 0.5, 1, and 1.5 mM formaldehyde were 1.4 ± 0.02, 1.0 ± 0.03, 0.75 ± 0.01, 0.33 ± 0.01, and 0.16 ± 0.01 h^–1^, respectively (Supplementary Table S4). Comparatively, the growth rates of the plasmid-cured evolved clone in the presence of 0, 0.25, 0.5, 1, and 1.5 mM formaldehyde were 1.2 ± 0.00, 1.1 ± 0.01, 0.76 ± 0.00, 0.34 ± 0.00, and 0.18 ± 0.00 h^–1^, respectively (Supplementary Table S4). The slight growth defect observed in the plasmid-cured evolved clone compared to the plasmid-cured parental strain likely results from genomic mutations developed during chemical mutagenesis and directed evolution. These mutations are discussed in the WGS section below.

### Activities of the Methylotrophic Enzymes Are Retained Following Chemical Mutagenesis and Adaptive Laboratory Evolution

**FIGURE 3:**
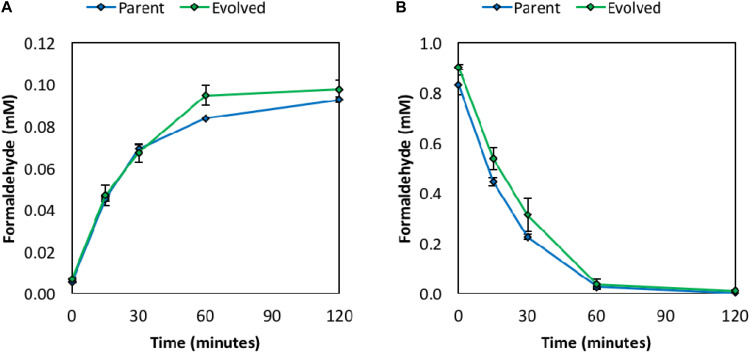
Formaldehyde production from 1 M methanol *in vivo* **(A)** and formaldehyde consumption from 1 mM formaldehyde *in vivo* **(B)** in resting cells in M9 minimal medium. Error bars indicate standard error (*n* = 2).

To investigate whether the improved methanol tolerance of the evolved clone results from improved rates of methanol and/or formaldehyde consumption via the Mdh and RuMP pathway enzymes, *in vivo* formaldehyde production and consumption assays were performed. Briefly, resting cells in minimal medium were used to monitor formaldehyde production following the addition of 1 M methanol (Figure 3A) or formaldehyde consumption following the addition of 1 mM formaldehyde (Figure 3B). The rates of formaldehyde production and consumption were similar between the parental strain and evolved clone, suggesting that the *in vivo* activities of the methylotrophic enzymes (Mdh, Hps, and Phi) are retained following chemical mutagenesis and ALE. Retention of *in vivo* activities of the methylotrophic enzymes, and thus a functional synthetic methanol utilization pathway, is further confirmed as methanol-derived carbon is still assimilated into intracellular metabolites following ALE, which is discussed in more detail in the methanol assimilation section below. These results are supported by WGS analysis (discussed in the WGS section below), which did not reveal any unique mutations in the pUD9 plasmid following chemical mutagenesis and ALE. Thus, it does not appear that the improved methanol tolerance of the evolved clone results from an increased rate of methanol and/or formaldehyde consumption through the synthetic methanol utilization pathway. Therefore, chromosomal mutations appear responsible for the improved methanol tolerance of the evolved clone, which agrees with the previous studies in *C. glutamicum* (Lessmeier and Wendisch, 2015; Wang et al., 2020).

### Improved Methanol Tolerance Can Readily Be Achieved in Other *E. coli* Strains, Including Those That Are Non-methylotrophic

We previously examined mutants of several transcriptional regulators and found that deletion of integration host factor subunit α (*ihfA*), which is known to repress multiple amino acid metabolic pathways (Goosen and van de Putte, 1995; Karp et al., 2018), resulted in an improved methylotrophic phenotype, as indicated by increased ^13^C-labeling of intracellular metabolites from ^13^C-methanol (Bennett et al., 2020a). Overexpression of cAMP-receptor protein (*crp*), a known activator of multiple amino acid metabolic pathways (Karp et al., 2018), also resulted in an improved methylotrophic phenotype (Bennett et al., 2020a). Given the success of ALE in improving the methanol tolerance of the synthetic *E. coli* methylotroph described above (*E. coli* BW25113 Δ*frmA* + pUD9), we sought to also improve the methanol tolerance of these transcriptional regulator mutant strains. *E. coli* Δ*frmA*Δ*ihfA* + pUD9 (simply referred to as “Δ*ihfA*”) and Δ*frmA* + pUD9 + pCrp (simply referred to as “pCrp”) were serially passaged in LB medium supplemented with increasing methanol concentrations in a similar manner as before (Supplementary Figure S6). However, chemical mutagenesis was not used for this ALE. Once tolerance to 2 M methanol was achieved, which required ca. 10–15 passages, six clones of each strain were examined for improved growth in 2 M methanol as compared to the respective parental strains (Supplementary Figure S7). The “Evolved 2” clone from each strain, hereafter referred to as “evolved pCrp” and “evolved Δ*ihfA*,” exhibited the most improved methanol tolerance and were selected for WGS analyses to identify common mutations responsible for methanol tolerance.

To assess whether a non-methylotrophic *E. coli* strain could achieve improved methanol tolerance, we serially passaged *E. coli* Δ*frmA* (containing no plasmid) in LB medium supplemented with increasing methanol concentrations in a similar manner as before without chemical mutagenesis. After 11 passages, isolates were examined for improved methanol tolerance in 2 M methanol (Supplementary Figure S8). Interestingly, this non-methylotrophic *E. coli* strain did achieve improved methanol tolerance through ALE, suggesting that methanol tolerance is not specific to methylotrophic strains. The “KB201” clone (Supplementary Figure S8B), an evolved, non-methylotrophic *E. coli* BW25113 Δ*frmA* strain (containing no plasmid), was selected for WGS analyses to identify common mutations responsible for methanol tolerance.

### Whole Genome Sequencing Revealed Common Mutations Responsible for Methanol Tolerance

We next sought to determine whether the evolved strains accumulated common genomic mutations that contributed to the methanol tolerance phenotype. We performed WGS analysis of the original evolved strain (Δ*frmA* + pUD9), KB201 and the evolved Δ*ihfA* clone. The evolved pCrp clone was excluded from WGS analysis for simplicity. Each evolved strain accumulated multiple unique mutations when compared to the respective parental strains (Supplementary Table S5). Of considerable interest were mutations found in 30S ribosomal subunit proteins, which were common among all of the evolved strains. Both the evolved Δ*ihfA* clone and KB201 had an identical mutation in *rpsQ*, a 30S ribosomal subunit protein S17, that resulted in a His31Pro change. The original evolved strain (Δ*frmA* + pUD9) had a point mutation in another 30S ribosomal subunit protein S12, *rpsL*, which resulted in a Gly92Ser amino acid change. The original evolved strain (Δ*frmA* + pUD9) was also observed to have a larger number of mutations, especially transition mutations, due to chemical mutagenesis prior to ALE. Since the only common mutation occurring in all three evolved strains was specific to a 30S ribosomal subunit protein, we hypothesize that methanol tolerance results from increased translational efficiency (Haft et al., 2014). To support this hypothesis, an identical mutation in *rpsQ* (H31P) was found in a previous study that examined ethanol tolerance of *E. coli* (Haft et al., 2014). This mutation was found to protect cells from ethanol toxicity by increasing the accuracy of protein synthesis. This suggests that the mechanisms of methanol and ethanol tolerance in *E. coli* are similar and not specific to methylotrophic metabolism, which is why methanol tolerance in the non-methylotrophic *E. coli* strain was readily achieved. Compared to previous methanol tolerance studies, these results provide a novel insight into alternative mechanisms of methanol tolerance.

### Improved Methanol Tolerance Leads to Enhanced Synthetic Methanol Assimilation

**FIGURE 4:**
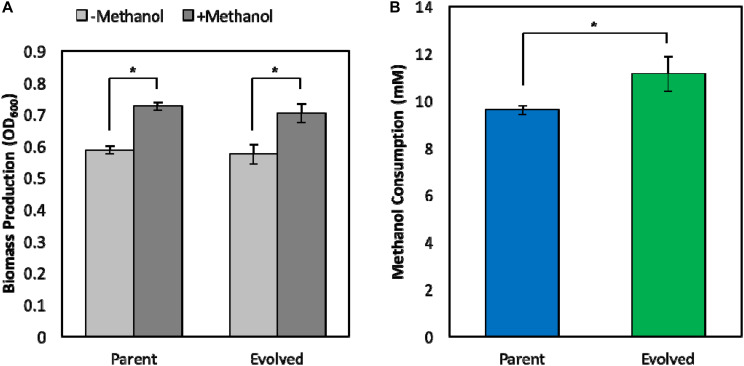
Phenotypic characterization of parental and evolved methylotrophic *E. coli* strains. **(A)** Total biomass production during 48 h growth in M9 minimal medium supplemented with 1 g/L yeast extract in the absence (–Methanol) or presence (+Methanol) of 60 mM ^13^C-methanol. **(B)** Total methanol consumption over the course of 48 h. Error bars indicate standard deviation (*n* = 3). *p < 0.05.

Improving the methanol tolerance of a synthetic *E. coli* methylotroph is not beneficial unless the evolved clone retains the methylotrophic phenotype, i.e., growth on methanol, at low, non-toxic methanol concentrations, which are more practical for industrial bioprocesses to minimize substrate loss via evaporation and ensure complete substrate utilization. Previously, we demonstrated, for the first time, that a synthetic *E. coli* methylotroph is capable of growth on methanol with a small amount of yeast extract supplementation (Whitaker et al., 2017). To ensure that the original evolved strain (Δ*frmA* + pUD9) still exhibits the parental methylotrophic growth phenotype, methylotrophic growth assays in minimal medium supplemented with 1 g/L of yeast extract in the absence and presence of 60 mM ^13^C-methanol were performed. Upon yeast extract exhaustion, we previously demonstrated that methylotrophic *E. coli* is able to grow on methanol for a brief period, resulting in improved biomass production and termed “methanol boost” (Whitaker et al., 2017). Here, we determined the methylotrophic characteristics of the original evolved strain (Δ*frmA* + pUD9) and compared them with the respective parental strain. Cultures were inoculated to an OD_600_ of approximately 0.05, and samples were collected at 48h for determination of biomass production, methanol consumption and ^13^C-labeling. Both strains exhibited similar methylotrophic growth phenotypes in terms of biomass production as the total biomass production in the presence of methanol was approximately 25% higher than that in the absence of methanol, demonstrating that both strains exhibit growth on methanol and a methanol boost of ca. 25% (Figure 4A). Although biomass production profiles were similar between the two strains, total methanol consumption was improved in the original evolved strain (Δ*frmA* + pUD9), which consumed 11.1 ± 0.7 mM methanol over the course of 48 h, representing a 16% increase in total methanol consumption over the parental strain, which consumed 9.6 ± 0.2 mM methanol over the course of 48 h (Figure 4B).

**FIGURE 5:**
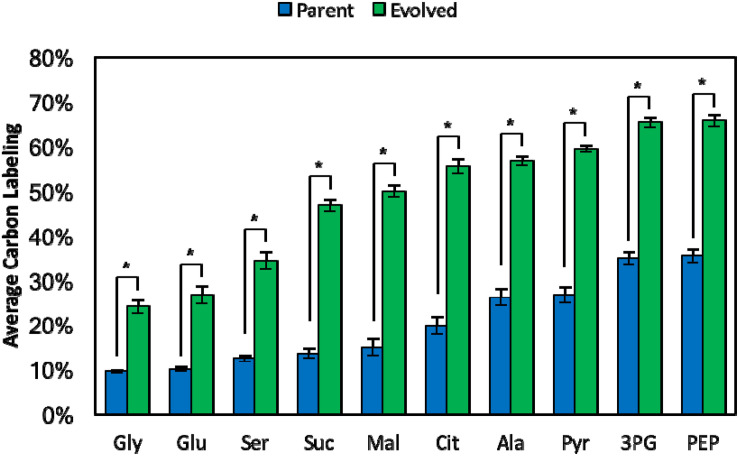
Average carbon labeling of intracellular metabolites and amino acids at 48 h from parental and evolved methylotrophic *E. coli* strains grown in M9 minimal medium supplemented with 1 g/L yeast extract and 60 mM ^13^C-methanol. Intracellular metabolites and amino acids: glycine (Gly), glutamate (Glu), serine (Ser), succinate (Suc), malate (Mal), citrate (Cit), alanine (Ala), pyruvate (Pyr), 3-phosphoglycerate (3PG), phosphoenolpyruvate (PEP). Error bars indicate standard deviation (*n* = 3). *p < 0.05.

Although total methanol consumption was improved in the original evolved strain (Δ*frmA* + pUD9), we aimed to determine whether more methanol was assimilated into metabolites and biomass components since methanol consumption includes both assimilation into central metabolism and dissimilation to formate and CO_2_, even with Δ*frmA*. To quantify methanol assimilation, ^13^C-labeling in intracellular metabolites and proteinogenic amino acids, derived from ^13^C-methanol, was determined. Indeed, significantly higher ^13^C-lableing was realized in the original evolved strain (Δ*frmA* + pUD9) as compared to the respective parental strain (Figure 5). For example, average carbon labeling in pyruvate (Pyr), a lower glycolytic intermediate, was increased from 26.7 ± 1.9% in the parental strain to 59.4 ± 0.7% in the evolved clone, representing an increase of 120%. Additionally, average carbon labeling in citrate (Cit), a TCA cycle intermediate, was increased from 19.8 ± 2.0% in the parental strain to 55.5 ± 1.7% in the evolved clone, representing an increase of 180%. Finally, average carbon labeling in alanine (Ala), a proteinogenic amino acid derived from Pyr, was increased from 26.2 ± 2.0% in the parental strain to 56.8 ± 0.9% in the evolved clone, representing an increase of 120%. Taken together, these results suggest that the original evolved strain (Δ*frmA* + pUD9) not only consumes more methanol, but also assimilates more methanol-derived carbon into intracellular metabolites and proteinogenic amino acids, which is a crucial characteristic for industrial methanol bioprocesses. Though the evolved strain exhibited increased consumption and assimilation of ^13^C-methanol, the absolute amount of improved consumption was low (ca. 2 mM) compared to the parental strain. This low amount is insufficient to generate a substantial improvement in biomass production, but it provides a step forward toward autonomous methylotrophy as indicated by the ^13^C-labeling analysis. Furthermore, the ^13^C metabolite data represent relative, not absolute, labeling, which further supports why consumption and assimilation of methanol, but not biomass production, are improved in the evolved strain.

## Discussion

Significant progress has been made toward developing synthetic methylotrophs for industrial methanol bioconversion. However there are still limitations that must be alleviated prior to industrial implementation. Here, we focused on improving the methanol tolerance of synthetic *E. coli* methylotrophs via ALE. Improved methanol tolerance was acquired by several distinct strains following ALE, and WGS analysis revealed that a common mutation in 30S ribosomal subunit proteins was responsible for methanol tolerance. Specifically, mutations found in the *rpsL* and *rpsQ* genes, which encode 30S ribosomal subunit proteins S12 and S17, respectively, are responsible for the improved methanol tolerance phenotype in all evolved strains.

Certain mutations in *rpsL* are known to cause hyperaccurate (restrictive) translational phenotypes, and were first discovered in connection with streptomycin resistant phenotypes (Gorini and Kataja, 1964; Ozaki et al., 1969). Hyperaccurate phenotypes typically result from mutations to residues in the decoding interface between S12 and 16S rRNA, where tRNA selection occurs (Ogle et al., 2002; Zaher and Green, 2010; Demirci et al., 2013). The mutation in *rpsL*, Gly92Ser, is close to the decoding interface of the ribosome (Wimberly et al., 2000; Ogle et al., 2002). A study on ethanol tolerance mechanisms in *E. coli* found that a His31Pro mutation in *rpsQ*, which resulted from ALE in the presence of increasing ethanol concentrations, conferred protection to the cells via increased ribosomal accuracy, suggesting that the mechanisms of methanol and ethanol tolerance in *E. coli* are similar and not specific to methylotrophic metabolism. In the presence of 40 g/L ethanol, a strain harboring the *rpsQ* His31Pro mutation drastically reduced translational misreading to levels similar to that of the wild type strain grown without ethanol (Haft et al., 2014).

These findings provide a novel insight into methanol tolerance mechanisms in *E. coli* and other bacteria as earlier studies identified alternative mechanisms of methanol tolerance in *C. glutamicum*. In one study, it was determined that two point mutations, (A165T in O-acetylhomoserine sulfhydrolase (MetY) and Q342^∗^ in CoA transferase (Cat), were responsible for improving the methanol tolerance of a wild-type *C. glutamicum* strain. The enzymatic side reactions of MetY and Cat were found to contribute to methanol toxicity as MetY catalyzes the alkylation of O-acetylhomoserine with methanol to generate acetate and O-methylhomoserine, which inhibits bacterial growth (Lessmeier and Wendisch, 2015), and Cat acts as a potential acetyl-CoA hydrolase, alcohol acetyltransferase or for the generation of methyl-CoA, potentially generating intermediates that also inhibit growth (Lessmeier and Wendisch, 2015). A separate study found that two mutations were crucial for improving the methanol tolerance of a methylotrophic *C. glutamicum* methanol auxotroph (Tuyishime et al., 2018; Wang et al., 2020). One mutation was found in MetY, similar to the prior *C. glutamicum* study, and another mutation was found in a methanol-induced membrane-bound transporter. Taken together, these results highlight the many direct and indirect effects of methanol toxicity and various ways to achieve improved methanol tolerance.

Importantly, for the context of synthetic methylotrophy, the original evolved strain (Δ*frmA* + pUD9) exhibited improved methanol consumption and assimilation of methanol-derived carbon into intracellular metabolites and proteinogenic amino acids, highlighting the unique ability to couple improved methanol tolerance with enhanced synthetic methanol utilization. Overall, this study represents a step forward in the field of synthetic methylotrophy by providing novel insights into methanol tolerance mechanisms and strategies to improve methanol bioconversion without the need for rational engineering.

## Data Availability Statement

The datasets presented in this study can be found in online repositories. The names of the repository/repositories and accession number(s) can be found below: BioSample accessions SAMN17309453, SAMN17309454, and SAMN17309455.

## Author Contributions

RB, GG, and EP designed the research, analyzed the data, and wrote the manuscript. RB, GG, JG, and JH conducted the experiments. All authors read and approved the manuscript.

## Conflict of Interest

The authors declare that the research was conducted in the absence of any commercial or financial relationships that could be construed as a potential conflict of interest.
