# Supplementary material for: Improving the Methanol Tolerance of an Escherichia coli Methylotroph via Adaptive Laboratory Evolution Enhances Synthetic Methanol Utilization
Source: Front Microbiol. 2021 Feb 11;12:638426. doi: 10.3389/fmicb.2021.638426 (PMC7904680; doi:10.3389/fmicb.2021.638426)
Supplement: Supplementary file 1 [file Data_Sheet_1.pdf]

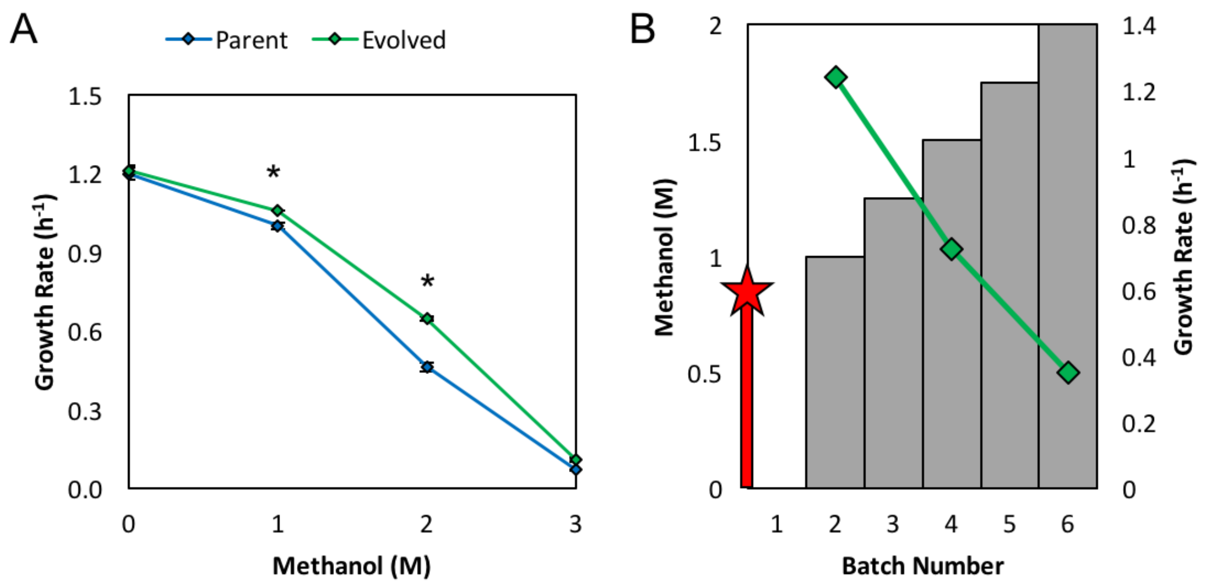

Fig. S1. (A) Maximum growth rate of parent and evolved methylotrophic *E. coli* in LB medium supplemented with methanol at the specified concentrations. (B) Chemical mutagenesis and directed evolution. NTG treatment (red star) was followed by overnight recovery in LB medium in the absence of methanol (batch 1). Serial passaging was then performed in fresh LB medium supplemented with increasing methanol concentrations (batches 2-6). The growth rate of cultures during the ALE process is represented by the green line. Error bars indicate standard error (n=2). *, $p < 0.05$.

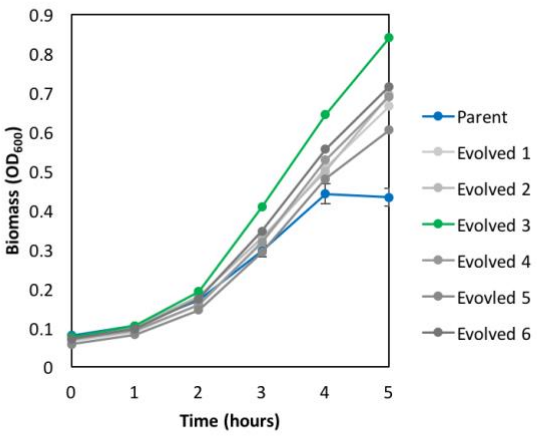

Fig. S2. Growth of isolated evolved clones in LB medium supplemented with 2 M methanol.

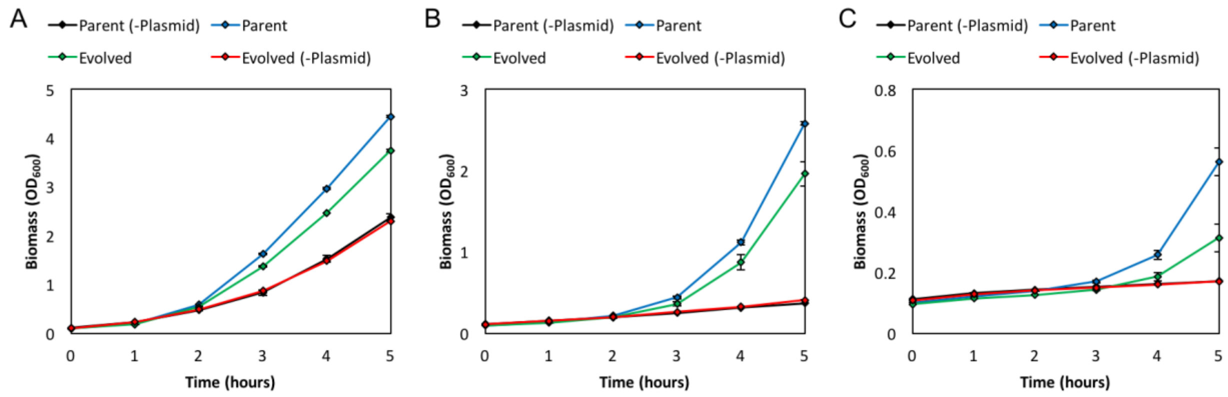

Fig. S3. Growth of plasmid-cured and plasmid-intact parent and evolved *E. coli* strains in LB medium supplemented with 0.5 (A), 1 (B) or 1.5 (C) mM formaldehyde.

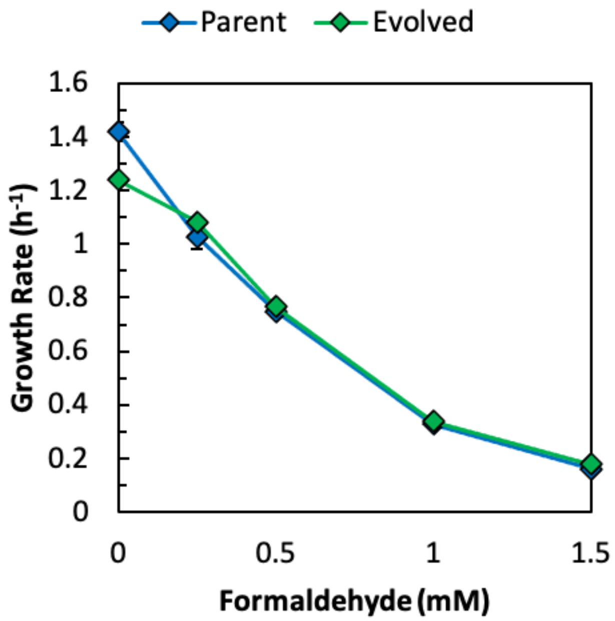

Fig. S4. Maximum growth rate of plasmid-cured parent and evolved *E. coli* in LB medium supplemented with formaldehyde at the specified concentration. Error bars indicate standard error (n=2).

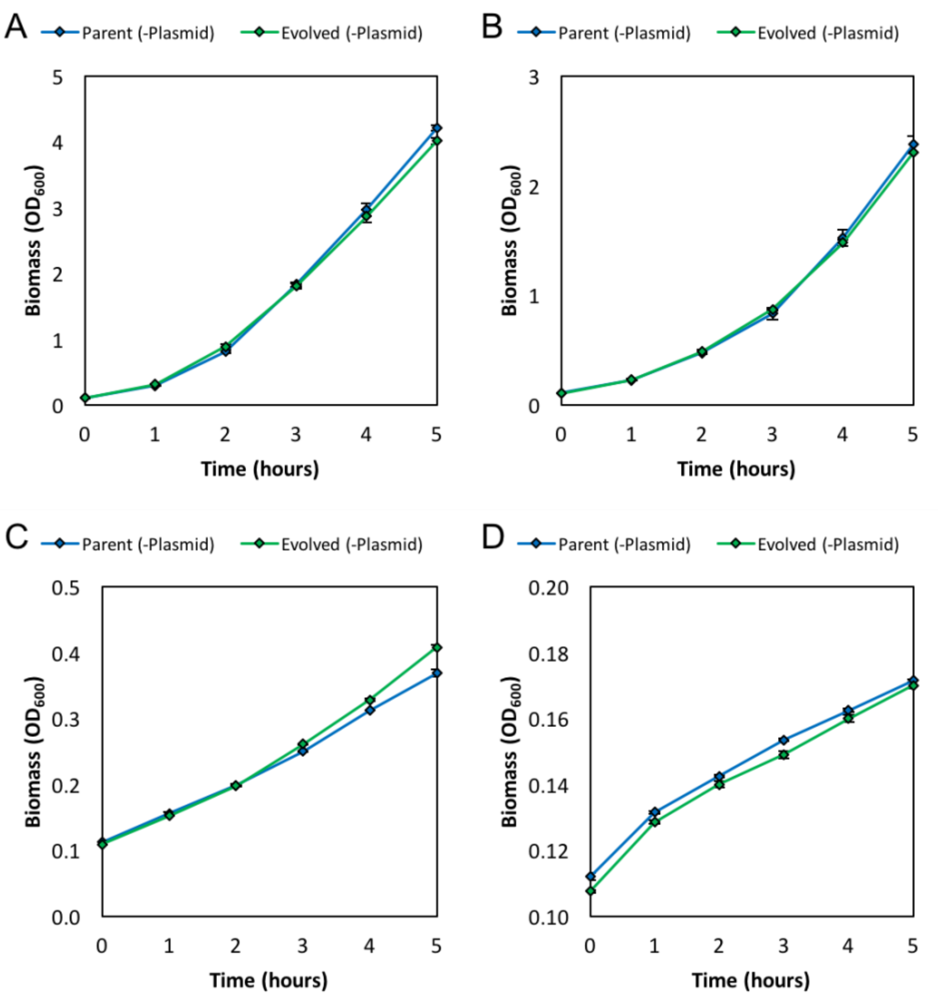

Fig. S5. Growth of plasmid-cured parent and evolved *E. coli* strains in LB medium supplemented with 0.25 (A), 0.5 (B), 1 (C) or 1.5 (D) mM formaldehyde.

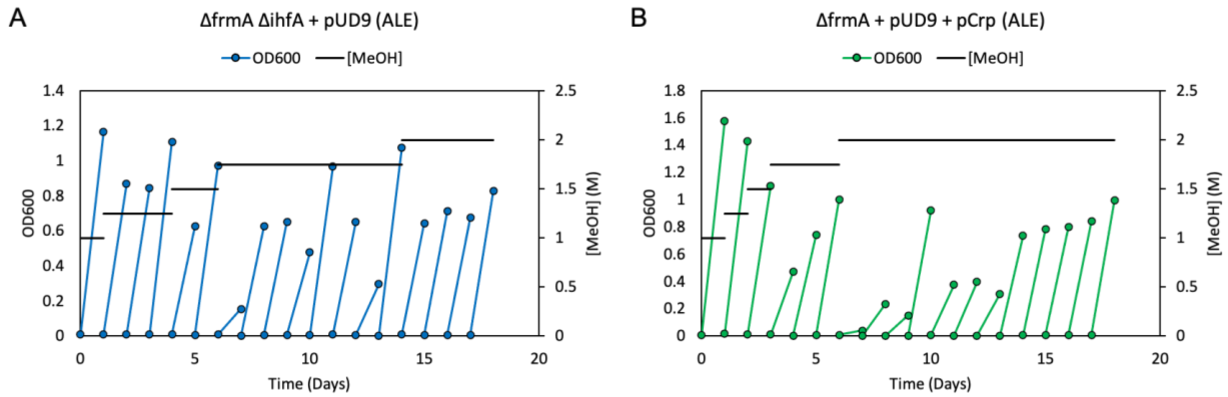

Fig. S6. Evolutionary trajectory of *E. coli* Δ*frmA*Δ*ihfA*+pUD9 (A) and Δ*frmA*+pUD9+pCrp (B) in LB medium with increasing methanol concentrations.

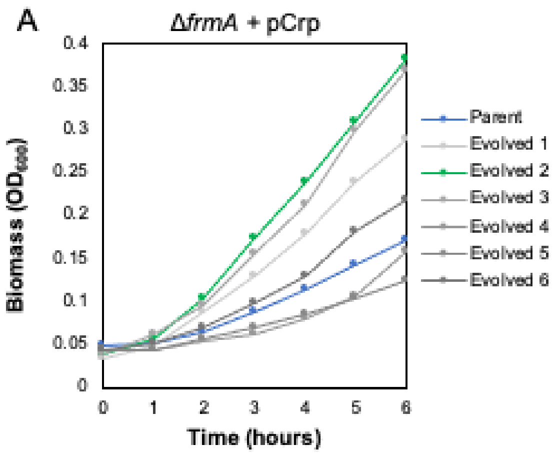

Fig. S7. Growth of isolated evolved clones of the pCrp overexpression strain (A) and the Δ*ihfA* knockout strain (B) in LB medium supplemented with 2 M methanol.

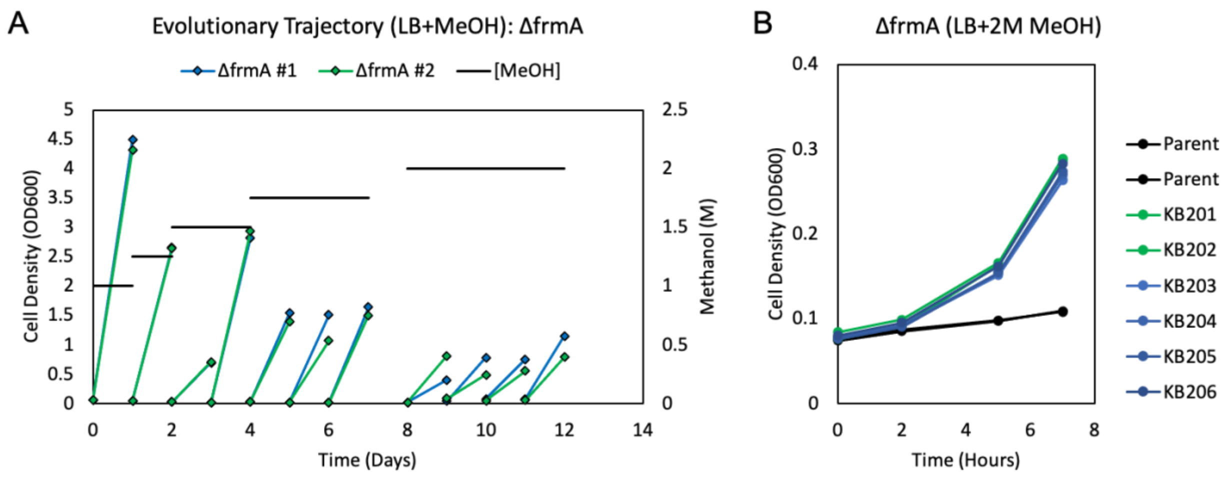

Fig. S8. Evolutionary trajectory of *E. coli* Δ*frmA* in LB medium with increasing methanol concentrations (A) and isolation of methanol-tolerant clones in LB medium supplemented with 2 M methanol (B).
