# Supplementary material for: Improving the Methanol Tolerance of an Escherichia coli Methylotroph via Adaptive Laboratory Evolution Enhances Synthetic Methanol Utilization
Source: Front Microbiol. 2021 Feb 11;12:638426. doi: 10.3389/fmicb.2021.638426 (PMC7904680; doi:10.3389/fmicb.2021.638426)
Supplement: Supplementary file 2 [file Data_Sheet_2.pdf]

Table S1. Strains used in this study.

**Name**	**Strain**	**Genotype**	**Source**
NEB5a	*E. coli* NEB5a	*fhuA2 Δ(argF-lacZ)U169 phoA glnV44 Φ80 Δ(lacZ)M15 gyrA96 recA1 relA1 endA1 thi-1 hsdR17*	NEB (Ipswich, MA)
BW25113	*E. coli* BW25113	*Δ(araD-araB)567 ΔlacZ4787(::rrnB-3) λ- rph-1 Δ(rhaD-rhaB)568 hsdR514*	Baba et al., 2006
ΔfrmA	*E. coli* BW25113 D*frmA*	BW25113 D*frmA::kan*	Whitaker et al., 2017
ΔfrmA ΔihfA	*E. coli* BW25113 D*frmA* *ΔihfA*	BW25113 D*frmA::FRT* *ΔihfA::kan*	Bennett et al., 2020a

Table S2. Plasmids used in this study.

**Name**	**Plasmid**	**Characteristics**	**Source**
pKD13	pKD13	*R6K ori, bla, kan, FRT*	Datsenko and Wanner, 2000
pKD46	pKD46	*oriR101, repA101(ts), bla, araB,* ǀ *red bet,* ǀ *red gam,* ǀ *red exo*	Datsenko and Wanner, 2000
pCP20	pCP20	*repA101(ts), cI857(ts), bla, cat, S. cerevisiae flp*	Datsenko and Wanner, 2000
pETM6	pETM6	*ColE1 ori, bla, f1 ori, lacI, T7 MCS*	Xu et al., 2012
pUD9	pETM6_P$_{trc}$_*mdh* _*hps* _*phi*	pETM6, *Ptrc, B. stearothermophilus mdh, B. methanolicus hps, B. methanolicus phi*	Bennett et al., 2018a
pACM4	pACM4	*P15A ori, cat, f1 ori, lacI, T7 MCS*	Xu et al., 2012
pCrp	pACM4_P$_{tac}$_*crp*	pACM4, *Ptac* , *E. coli* BW25113 *crp*	Bennett et al., 2020a

Table S3. Primers used in this study.

Primer	Sequence (5' to 3')	Purpose
ihfA KO F	AGAGGCATTAAAAGAGCGATTCCAGGCATCATTGAGGGATTGAACCTATGATTCCGGGGATCCGTCGACC	Gene knockout
ihfA KO R	CGCAGAGCGGCCTTTTTAGTTAGATCAGATTACTCGTCTTTGGGCGAAGCTGTAGGCTGGAGCTGCTTCG	
ihfA F	GGATGCCAAAGAACTGGTTG	Gene-specific confirmation
ihfA R	CACGCAGATCGAAGTTACCA	
ihfA up F	TTGGTTTTGTTGGGGTTGTT	Locus-specific confirmation
ihfA down R	TGAAGTGTCATGGCGTTGAT	
kan F	TGAATGAACTGCAGGACGAG	Gene-specific confirmation
kan R	AATATCACGGGTAGCCAACG	
pACM4 F Gib	TCGAGTCTGGTAAAGAAAC	PCR for Gibson assembly
pACM4 R Gib	TGTATATCTCCTTCTTAAAGTTAAAC	
pACM4 up F	CGACTCCTGCATTAGGAAGC	Sequencing confirmation
pACM4 down R	GGCAGTTATTGGTGCCCTTA	
crp F pAC Gib	TTTAACTTTAAGAAGGAGATATACAATGGTGCTTGGCAAACCG	PCR for Gibson assembly
crp R pAC Gib	GCAGCGGTTTCTTTACCAGACTCGATTAACGAGTGCCGTAAACG	

Table S4. Maximum growth rates of parent and evolved *E. coli* strains in LB medium supplemented with methanol or formaldehyde at the specified concentrations. Data represent average ± standard error (n=2).

	+ Plasmid		- Plasmid	
	**Parent**	**Evolved**	**Parent**	**Evolved**
**Methanol (M)**	**Growth Rate (h^{-1})**			
**0**	1.2 ± 0.02	1.2 ± 0.02	1.4 ± 0.02	1.2 ± 0.00
**1**	1.0 ± 0.01	1.1 ± 0.00	1.3 ± 0.00	1.2 ± 0.01
**2**	0.47 ± 0.02	0.65 ± 0.01	0.66 ± 0.00	0.66 ± 0.01
**3**	0.07 ± 0.00	0.11 ± 0.01	0.18 ± 0.00	0.15 ± 0.00
**Formaldehyde (mM)**	**Growth Rate (h^{-1})**			
**0.25**	-	-	1.0 ± 0.03	1.1 ± 0.01
**0.5**	-	-	0.75 ± 0.01	0.76 ± 0.00
**1**	-	-	0.33 ± 0.01	0.34 ± 0.00
**1.5**	-	-	0.16 ± 0.01	0.18 ± 0.00

Table S5. Unique sequence variants of evolved clones determined via whole genome sequence with *E. coli* BW25113 as the reference genome. I=insertion, D=deletion, nucleotide changes are indicated by '→'. Cells highlighted in blue indicate common mutations in tRNAs. Cells highlighted in yellow indicate common mutations in 30S ribosomal subunit proteins.

Strain	Position in BW25113 genome	Nucleotide change (+ strand)	Amino acid change	Gene
KB201	1011663	C→G	Trp93Cys	substitution in *fabA*, beta dehydroxydecanoyl thioester dehydrase
	3033130	T→A	n/a	substitution in *xerD* promoter
	3441364	G→A	Arg49Cys	substitution in *rplN*, 50S ribosomal subunit
	3441836	T→G	His31Pro	substitution in *rpsQ*, 30S ribosomal subunit
	4210706	large insertion	n/a	large insertion in *arpA*, ankyrin repeat protein
Evolved Δ*ihfA* Clone	86049	DT	n/a	deletion in *mraZ* promoter, methyltransferase inhibitor
	219445	DA	frameshift	deletion in *gmhB*, D,D-heptose 1,7-bisphosphate phosphatase
	2434696	C→G	Gly130Ala	substitution in *fabB*, 3-oxoacyl-synthase I
	3441836	T→G	His31Pro	substitution in *rpsQ*, 30S ribosomal subunit
Original Evolved Strain (Δ*frmA*+pUD9)	557147	DG	frameshift	deletion in *sfmD*, putative outer membrane protein
	1415901	DC	frameshift	deletion in cryptic prophage Rac conserved protein *ydaU*
	1584807	G→A	n/a	substitution in *yneL* pseudogene promoter
	1632320	G→A	Ala16Val	substitution in *ynfN*, cold-shock protein in Qin prophage
	1763729	DG	frameshift	deletion in *ydiK*, inner membrane protein
	2196843	DG	frameshift	deletion in *yehI*, putative regulator
	2398891	DC	n/a	deletion upstream of *nuoA*, oxidoreductase, membrane subunitA
	3456961	C→T	Pro227Ser	substitution in *gspK*, transport protein
	3461340	C->T	Ala625Thr	substitution in *chiA*, periplasmic endochitinase
	3467638	C→T	Gly92Ser	substitution in *rpsL*, 30S ribosomal subunit
	3498571	C→T	Ser270Asn	substitution in *yhfS*, aminotransferase family protein
	3528705	C→T	Ala175Thr	substitution in *envZ*, sensor histidine kinase for *ompC* and *ompF*
	3721471	DC	frameshift	deletion in *xylB*, xylulokinase
	4328920	G→A	silent	substitution in *adiA*, arginine decarboxylase
	4596140	DG	n/a	deletion in *leuQ*, tRNA leucine
